# Improved Voigt and Reuss Formulas with the Poisson Effect

**DOI:** 10.3390/ma15165656

**Published:** 2022-08-17

**Authors:** Yunhua Luo

**Affiliations:** Department of Mechanical Engineering, University of Manitoba, Winnipeg, MB R3T 2N2, Canada; yunhua.luo@umanitoba.ca

**Keywords:** the Poisson effect, iso-strain and iso-stress conditions, Voigt and Reuss formulas, effective elastic properties, secondary strains and stresses

## Abstract

The Poisson effect, measured by the Poisson’s ratio, plays an important role in the regulation of the elastic properties of composite materials, but it is not considered in the conventional Voigt (iso-strain) and Reuss (iso-stress) formulas, which explains why the formulas are inaccurate even if the iso-strain or the iso-stress conditions are satisfied. To consider the Poisson effect, we derived a set of new formulas based on the governing equations of elasticity. The obtained formulas show greater mathematical complexity. To further understand how the Poisson effect influences composite elastic properties, two types of finite element models (FEM) were constructed to simulate the situations where the Poisson effect does or does not have an influence. The results show that the conventional Voigt and Reuss formulas are special cases of the newly derived ones. The Poisson effect induces secondary strains and stresses into the phase materials, which demands more strain energy to achieve the same deformation in the primary (loading) direction, and thus increases composite stiffness; the magnitude of the increase is dependent on the contrast of phase properties. The findings may have significant impact on the study of the emerging nanocomposites and functionally graded materials, where the conventional Voigt and Reuss formulas have wide applications.

## 1. Introduction

The Voigt and Reuss formulas [1,2], also known as the rule of mixtures and the inverse rule of mixtures in the literature, are the oldest and the simplest equations for the estimation of composite elastic properties. The formulas are derived from the iso-strain and the iso-stress assumptions, respectively, which represent the two extreme scenarios that the phase materials work together. Under the iso-strain condition, the phase materials work in parallel to achieve the maximum stiffness; while under the iso-stress condition, the phase materials work in serial to attain the maximum compliance or flexibility. The actual situation in a particulate composite is somewhere between the two extremes.

The Voigt and Reuss formulas, as well as their modified versions, are widely used for estimation of the upper and lower bounds of composite elastic properties [3,4] and for prediction of the elastic moduli of unidirectionally reinforced composites [5,6,7,8]. They also have applications in the study of emerging composites such as nanocomposites [9,10,11,12,13,14,15,16] and functionally graded materials [17,18,19,20], mainly due to their simplicity. For the unidirectionally reinforced composites, if the loading is applied only either in the longitudinal or in the transverse direction, the iso-strain or the iso-stress conditions are seemingly satisfied; therefore, the formulas should be accurate for the prediction of the respective modulus. Nevertheless, the predictions are inaccurate, especially for the transverse modulus. There exist a few discrepancies that are probably responsible for the inaccuracy. First, the Voigt and Reuss formulas treat the elastic properties, i.e., Young’s modulus, shear modulus, bulk modulus and Poisson’s ratio, as completely independent parameters, but they are actually related to each other via the elasticity relations. Second, in the Voigt and Reuss models, phase materials are assumed perfectly bonded to each other at their interface, and the Poisson effect is not considered. However, the Poisson effect induces lateral strains in the phase materials, at the bonded interface the phase strains are ‘forced’ to be the same. To the author’s best knowledge, the effect of the above discrepancies on the accuracy of the conventional Voigt and the Reuss formulas has not been fully studied.

In this paper, we first derive a set of new formulas, which are the counterparts of the Voigt and the Reuss formulas but with the Poisson effect considered. Then, we conduct a finite element investigation to understand how the Poisson effect influences composite elastic properties under the respective iso-strain and iso-stress conditions.

## 2. Methods

**Figure 1:**
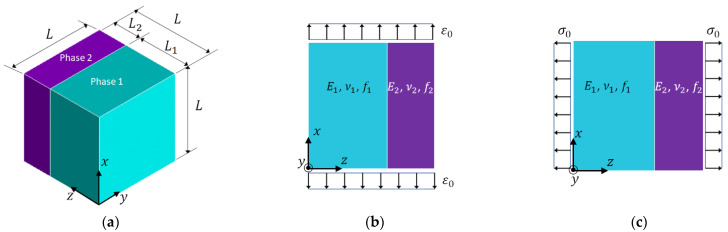
(**a**) Composite RVE; (**b**) iso-strain condition; (**c**) iso-stress condition.

To study the Poisson effect, let us consider the representative volume element (RVE) of a two-phase composite shown in Figure 1a, where the phase materials are represented by two blocks and their properties are described by the parameters shown in the figure. The symbols that describe the phase properties and phase variables are listed in Table 1. It is assumed that the phase materials are homogeneous and isotropic.

Under the iso-strain condition shown in Figure 1b, the effective Young’s modulus of the RVE in the x (or y) direction is determined by the Voigt formula [1],

(1) $${\bar{E}}_{\mathrm{Voigt}}={\bar{E}}_{x}={\bar{E}}_{y}=f_{1}E_{1}+f_{2}E_{2}$$

Under the iso-stress condition in Figure 1c, the effective Young’s modulus of the RVE in z direction is decided by the Reuss formula [2],

(2) $${\bar{E}}_{\mathrm{Reuss}}={\bar{E}}_{z}=\frac{E_{1}E_{2}}{f_{2}E_{1}+f_{1}E_{2}}$$

Equations (1) and (2) are also applied to calculate the effective shear modulus, bulk modulus and Poisson’s ratio, with the Young’s moduli replaced by the relevant properties. This practice actually assumes that the elasticity constants are independent to each other. It should be also noted that the phase Poisson’s ratios do not appear in either Equation (1) or Equation (2), implicitly assuming that phase Poisson’s ratios have no influence on the effective Young’s moduli.

To correct the above discrepancies, a set of new formulas of effective Young’s modulus and effective Poisson’s ratio under the iso-strain or the iso-stress conditions are derived, with the Poisson effect considered. The derivation starts with the governing equations of elasticity. The detailed derivation steps can be found in Appendix A. The resulting formulas for the iso-strain condition in Figure 1b are given in Equations (3a)–(3c),

(3a) $${\bar{E}}_{\mathrm{iso}-\mathrm{strain}}={\bar{E}}_{x}={\bar{E}}_{y}=\;\frac{\left[f_{1}\left(1-\nu_{2}\right)E_{1}+f_{2}\left(1-\nu_{1}\right)E_{2}\right]\cdot \left[f_{1}\left(1+\nu_{2}\right)E_{1}+f_{2}\left(1+\nu_{1}\right)E_{2}\right]}{f_{1}\left(1-\nu_{2}^{2}\right)E_{1}+f_{2}\left(1-\nu_{1}^{2}\right)E_{2}}$$

(3b) $${\bar{\nu }}_{\mathrm{iso}-\mathrm{strain}}^{P}={\bar{\nu }}_{xy}=\;\frac{\left[f_{1}\nu_{1}\left(1-\nu_{2}\right)+f_{2}\nu_{2}\left(1-\nu_{1}\right)\right]\cdot \left[f_{1}\left(1+\nu_{2}\right)E_{1}+f_{2}\left(1+\nu_{1}\right)E_{2}\right]}{f_{1}\left(1-\nu_{2}^{2}\right)E_{1}+f_{2}\left(1-\nu_{1}^{2}\right)E_{2}}$$

(3c) $${\bar{\nu }}_{\mathrm{iso}-\mathrm{strain}}^{T}={\bar{\nu }}_{xz}=\frac{f_{1}\nu_{1}\left(1-\nu_{2}^{2}\right)E_{1}+f_{2}\nu_{2}\left(1-\nu_{1}^{2}\right)E_{2}}{f_{1}\left(1-\nu_{2}^{2}\right)E_{1}+f_{2}\left(1-\nu_{1}^{2}\right)E_{2}}$$

and those for the iso-stress condition in Figure 1c are provided in Equations (4a) and (4b).

(4a) $${\bar{E}}_{\mathrm{iso}-\mathrm{stress}}={\bar{E}}_{z}=\;\frac{E_{1}E_{2}\left[f_{1}\left(1-\nu_{2}\right)E_{1}+f_{2}\left(1-\nu_{1}\right)E_{2}\right]}{E_{1}E_{2}\left[f_{1}^{2}\left(1-\nu_{2}\right)+f_{2}^{2}\left(1-\nu_{1}\right)\right]+f_{1}f_{2}\left[\left(1+\nu_{2}\right)\left(1-2\nu_{2}\right)E_{1}^{2}+4\nu_{1}\nu_{2}E_{1}E_{2}+\left(1+\nu_{1}\right)\left(1-2\nu_{1}\right)E_{2}^{2}\right]}\;$$

(4b) $${\bar{\nu }}_{\mathrm{iso}-\mathrm{stress}}={\bar{\nu }}_{zx}={\bar{\nu }}_{zy}=\;\frac{E_{1}E_{2}\left[f_{1}\left(1-\nu_{2}\right)\nu_{1}+f_{2}\left(1-\nu_{1}\right)\nu_{2}\right]}{E_{1}E_{2}\left[f_{1}^{2}\left(1-\nu_{2}\right)+f_{2}^{2}\left(1-\nu_{1}\right)\right]+f_{1}f_{2}\left[\left(1+\nu_{2}\right)\left(1-2\nu_{2}\right)E_{1}^{2}+4\nu_{1}\nu_{2}E_{1}E_{2}+\left(1+\nu_{1}\right)\left(1-2\nu_{1}\right)E_{2}^{2}\right]}\;$$

In Equations (3a)–(3c), (4a) and (4b), $\bar{E}$ and $\bar{\nu }$ represent the effective Young’s modulus and effective Poisson’s ratio, respectively; the subscripts ‘iso-strain’ and ‘iso-stress’ indicate the corresponding conditions; the subscripts x, y and z, or their combinations, refer to the coordinate axes shown in Figure 1a. It should be pointed out that, in Equations (3b) and (3c) two effective Poisson’s ratios, ${\bar{\nu }}_{\mathrm{iso}-\mathrm{strain}}^{P}$ (or ${\bar{\nu }}_{xy}$) and ${\bar{\nu }}_{\mathrm{iso}-\mathrm{strain}}^{T}$ (or ${\bar{\nu }}_{xz}$), are derived under the iso-strain condition, because when a deformation is induced in the x direction, the lateral deformations in the y direction and in the z  direction are different. The superscript P indicates that the lateral deformation is in the isotropic plane x-y; while the superscript T means that the lateral deformation is transverse to the isotropic plane. The Voigt formula can only predict one effective Poisson’s ratio. Furthermore, in Equations (3a)–(3c), (4a) and (4b), the effective Young’s modulus and effective Poisson’s ratio are dependent on the phase Young’s moduli and the phase Poisson’s ratios. It can be verified by mathematical operations, if $\nu_{1}=\nu_{2}$, Equation (3a) will degenerate to Equation (1); if $\nu_{1}=\nu_{2}=0$, Equation (4a) will become Equation (2). Therefore, the conventional Voigt and Reuss formulas, i.e., Equations (1) and (2), can be considered as special cases of Equations (3a) and (4a), respectively. It should be noticed that the conditions for Equation (4a) to degenerate to Equation (2) are more stringent than those from Equation (3a) to Equation (1).

**Figure 2:**
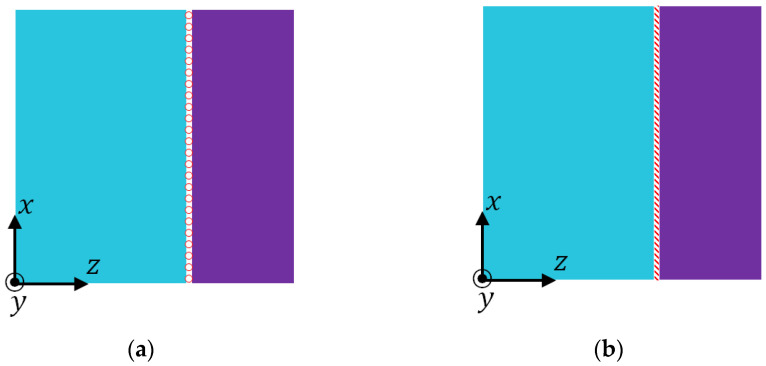
Models of phase interface. (**a**) Sliding; (**b**) bonded.

Although a comparison of the expressions in Equations (3a) and (4a) with those in Equations (1) and (2) shows the mathematical differences induced by the consideration of the Poisson effect, the underlying physical mechanism is still not clear. Therefore, a finite element study is conducted to understand how the Poisson effect influences the effective properties. In the finite element study, two models of the phase interface as shown in Figure 2 are considered. In the model shown in Figure 2a, the phase interface is sliding; while in Figure 2b, the phase interface is fully bonded.

The constraints on the displacements on the two sides of the interfaces are

(5) $$\mathrm{Sliding}\;\mathrm{Interface}\;\;\;u_{x}^{-}\neq u_{x}^{+},\;\;\;u_{y}^{-}\neq u_{y}^{+},\;\;\;u_{z}^{-}=u_{z}^{+}\;\mathrm{Bonded}\;\mathrm{Interface}\;\;\;u_{x}^{-}=u_{x}^{+},\;\;\;u_{y}^{-}=u_{y}^{+},\;\;\;u_{z}^{-}=u_{z}^{+}$$

where the superscripts ‘+’ and ‘–’ represent the two sides of the interface; the subscripts, x, y and z refer to the corresponding coordinate axes.

In addition to the two models of phase interface, two possible boundary conditions are applied onto the free surfaces of the RVE, one is homogeneous boundary conditions (HBC), the other is inhomogeneous boundary conditions (IBC). A free surface of the RVE has neither loading nor displacement constraint. In HBC, the finite element nodes on a free surface are forced to have the same displacement in the normal direction; while in IBC, the nodes are allowed to have different displacements in the normal direction. The boundary conditions for the implementation of the iso-strain condition in Figure 1b and the iso-stress condition in Figure 1c are described in Table 2.

In this finite element study, all quantities required for the determination of effective Young’s modulus and effective Poisson’s ratio of the RVE are computed using ANSYS Mechanical APDL (2020R2, ANSYS Inc., Canonsburg, PA, USA). RVE Young’s modulus (${\bar{E}}_{i}$) and Poisson’s ratio (${\bar{\nu }}_{ij}$) are determined from the average stresses (${\bar{\sigma }}_{i}$) and average strains (${\bar{\varepsilon }}_{i}$) by [20],

(6) $${\bar{E}}_{i}=\frac{{\bar{\sigma }}_{i}}{{\bar{\varepsilon }}_{i}}\;,\;\;\;\;(i=x,y,z)$$

(7) $${\bar{\nu }}_{ij}=-\frac{{\bar{\varepsilon }}_{j}}{{\bar{\varepsilon }}_{i}}\;,\;\;\;\;(i,j=x,y,z)$$

The average stresses and the average strains are calculated from the finite element stresses ($\sigma_{i}$) and strains ($\varepsilon_{i}$) as

(8) $${\bar{\sigma }}_{i}=\frac{1}{V}\int_{V}\sigma_{i}\;dV,\;\;\;\;{\bar{\varepsilon }}_{i}=\frac{1}{V}\int_{V}\varepsilon_{i}\;dV\;\;\;(i,j=x,y,z)$$

V is the volume of the RVE. Equation (8) can be also used to calculate average stresses in phase materials, simply with the RVE volume V replaced by the phase volumes $V_{1}$ and $V_{2}$.

Although a number of finite element models can be created from the combinations of the interface models and the types of boundary conditions, we mainly focus on the following two finite element models.

**FE Model I**: The sliding phase interface Figure 2a is combined with the IBC in Table 1. This model is expected to be consistent with Equations (1) and (2) and has no influence from the Poisson effect.

**FE Model II**: The bonded phase interface Figure 2b is combined with HBC in Table 1. This model is anticipated to agree with Equations (3a)–(3c) and (4a), (4b) and reflect the influence of the Poisson effect.

To investigate how the contrast of phase properties affects the influence of the Poisson effect, the four composites listed in Table 3 are studied, which represent different combinations of phase property contrasts. A property contrast is defined as the ratio between the higher value to the lower one.

The objective of this finite element study is to investigate differences between FE Model I and II in:

- The average phase stresses as calculated by Equation (8).
- The total strain energy in the RVE, which can be computed from the finite element stress and strain vectors, σ and ε, by $U=\frac{1}{2}\int_{V}\sigma \cdot \varepsilon \;dV$.
- The effective Young’s modulus and Poisson’s ratio as determined by Equations (6) and (7).

and ultimately to understand the physical mechanism that causes the differences between Equations (1) and (2) and Equations (3a) and (4a).

## 3. Results

To compare stresses in the two finite element models, the average phase stresses are distinguished into primary stress and secondary stresses. The primary stress is the normal stress in the loading direction, while the secondary stresses include all the other stress components. It was found that the fundamental difference between FE Model I and II is that no secondary stress is introduced for FE Model I no matter it is the iso-strain or the iso-stress condition, while secondary normal stresses are always induced for FE Model II. It should be mentioned that, for FE Model II, if the IBC is applied in replacement of HBC, the secondary stresses would also include shear stresses. The HBC are used because they are consistent with those considered in the derivation of Equations (3a)–(3c) and (4a), (4b).

**Figure 3:**
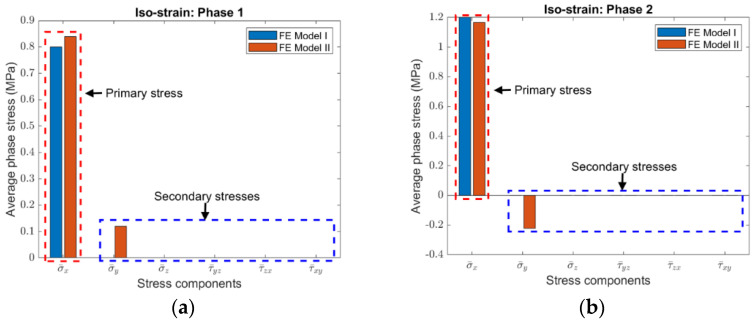
Average stresses in (**a**) Phase 1 and (**b**) Phase 2 of Composite #2 under the iso-strain condition (The volume fraction Phase 2 is 0.35).

**Figure 4:**
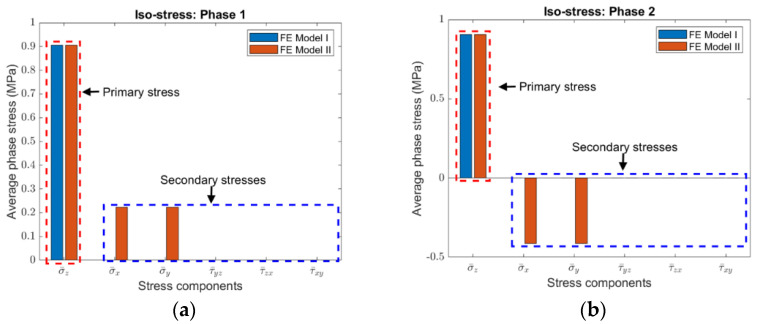
Average stresses in (**a**) Phase 1 and (**b**) Phase 2 of Composite #2 under the iso-stress condition (The volume fraction Phase 2 is 0.35).

Although the magnitudes of secondary stresses are different, the same phenomenon as described in the above was observed in the four composites with various volume fractions. As an example, the average phase stresses in Composite #2 under the iso-strain and the iso-stress conditions are plotted, respectively, in Figure 3 and Figure 4, the volume fraction of Phase 2 is 0.35.

**Figure 5:**
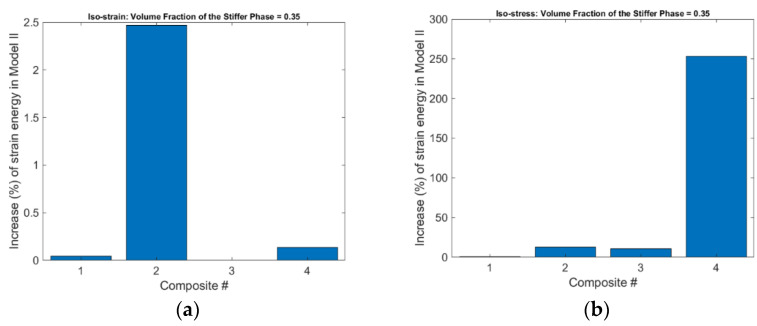
Increase (%) in strain energy in FE Model II compared with FE Model I to achieve the same deformation in the loading direction under (**a**) the iso-strain, and (**b**) the iso-stress conditions.

It was found that the total strain energy in FE Model II is always higher than that in FE Model I under the same conditions, no matter which composite and what volume fraction are simulated. The increases of strain energy in FE Model II compared with FE Model I in Composite #2 with a volume fraction of 0.35 are displayed in Figure 5, where the increase percentage is calculated as

$$\mathrm{Increase}\;\left(\%\right)\;\mathrm{of}\;\mathrm{Strain}\;\mathrm{Energy}=\frac{\mathrm{FE}\;\mathrm{Model}\;\mathrm{II}-\mathrm{FE}\;\mathrm{Model}\;I}{\mathrm{FE}\;\mathrm{Model}\;I}$$

**Figure 6:**
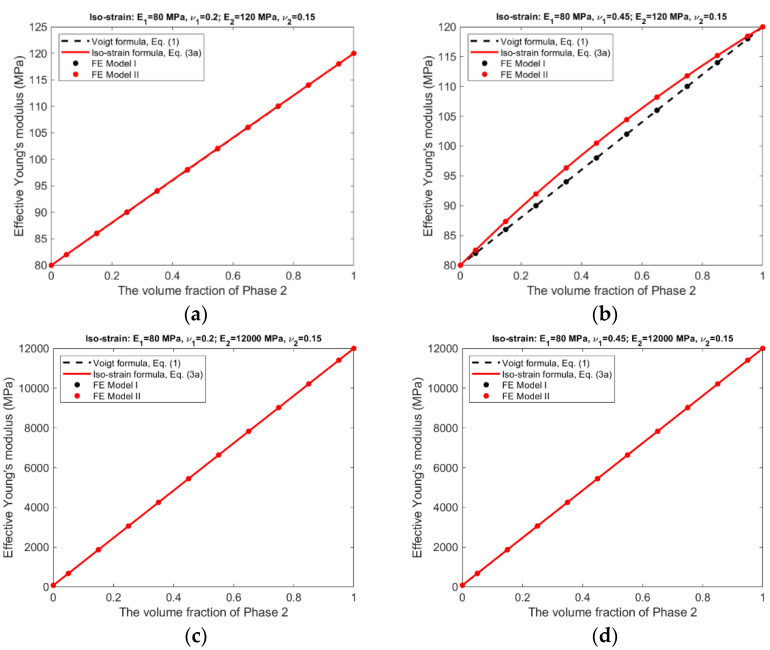
Iso-strain condition—Effective Young’s moduli predicted by analytical formulas and FEM. (**a**) Composite #1. (**b**) Composite #2. (**c**) Composite #3. (**d**) Composite #4.

**Figure 7:**
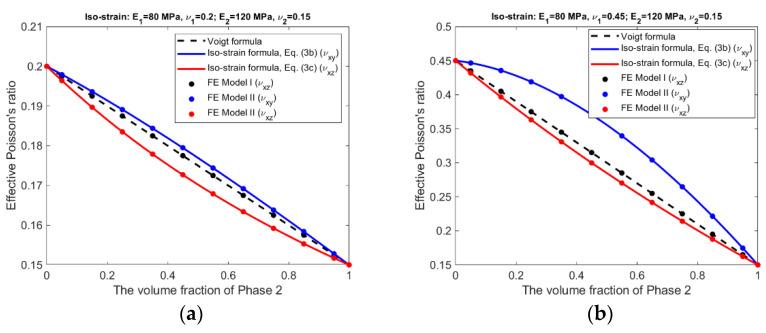

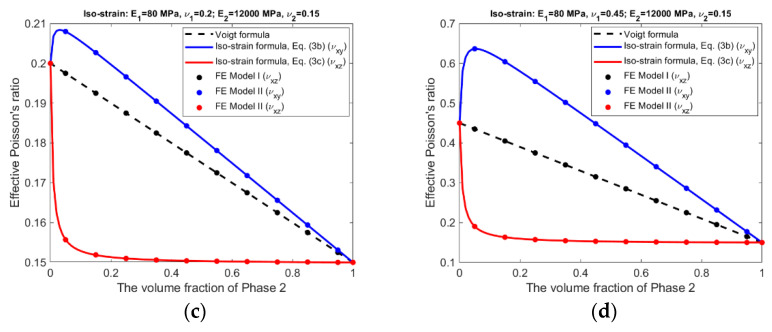
Iso-strain condition—Effective Poisson’s ratio predicted by analytical formulas and FEM. (**a**) Composite #1. (**b**) Composite #2. (**c**) Composite #3. (**d**) Composite #4.

For the iso-strain condition, the effective Young’s moduli and effective Poisson’s ratios predicted by the analytical formulas and the finite element modeling are presented in Figure 6 and Figure 7.

**Figure 8:**
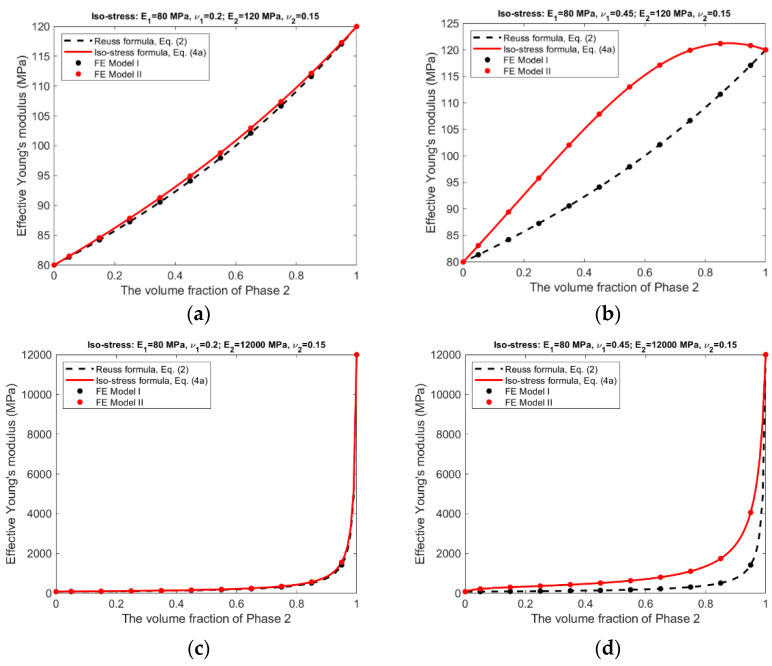
Iso-stress condition—Effective Young’s moduli predicted by analytical formulas and FEM. (**a**) Composite #1. (**b**) Composite #2. (**c**) Composite #3. (**d**) Composite #4.

**Figure 9:**
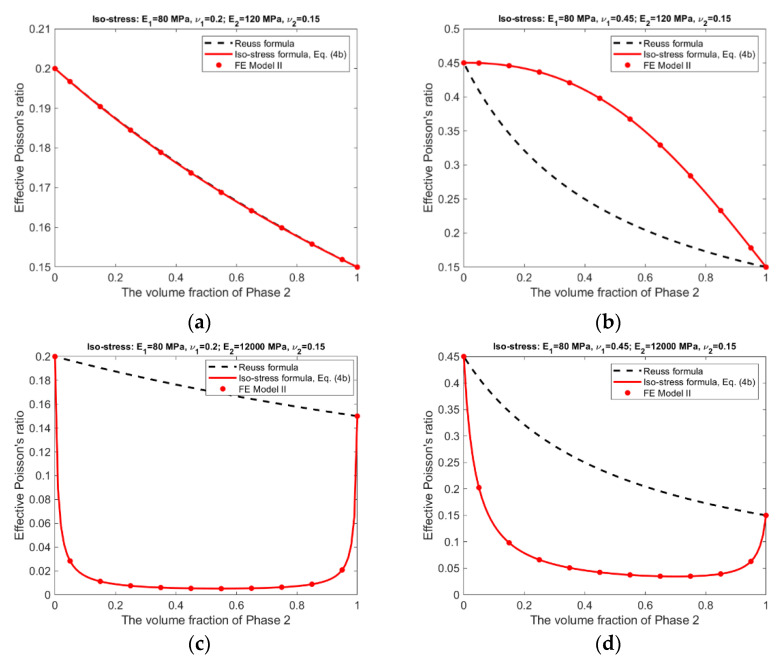
Iso-stress condition—Effective Poisson’s ratios predicted by analytical formulas and FEM. (**a**) Composite #1. (**b**) Composite #2. (**c**) Composite #3. (**d**) Composite #4.

For the iso-stress condition, the corresponding results are presented in Figure 8 and Figure 9.

It should be pointed out that for FE Model I, the effective Poisson’s ratio (${\bar{\nu }}_{xy}$) under the iso-strain condition and the effective Poisson’s ratios (${\bar{\nu }}_{zx}$ and ${\bar{\nu }}_{zy}$) under the iso-stress condition cannot be determined by the finite element modeling, because the phase materials have different lateral deformation and a simple averaging of the deformation is not able to produce meaningful results.

The following observations can be made from the results shown in Figure 6, Figure 7, Figure 8 and Figure 9.

- There is an excellent agreement between Equations (1) and (2) and FE Model I, and between Equations (3a)–(3c) and (4a), (4b) and FE Model II, suggesting that FE Model I and II do have the ability to simulate, respectively, the scenarios without and with the Poisson effect.
- The effective Young’s moduli predicted by either Equations (3a) and (4a) or FE Model II are always larger or at least equal to those by Equations (1) and (2) and FE Model I, cf. Figure 6 and Figure 8, indicating that the Poisson effect has the ability to increase composite stiffness.
- The Poisson effect has much greater influence over the effective Poisson’s ratio than over the effective Young’s modulus. The Voigt and the Reuss formulas generally have very low accuracy if they are applied to estimate the effective Poisson’s ratio.
- The Poisson effect has much greater influence over the effective properties under the iso-stress condition than under the iso-strain condition, compare Figure 8 and Figure 9 vs. Figure 6 and Figure 7.

## 4. Discussion

Based on the results presented in the previous section, the physical mechanism that introduces differences between Equations (1) and (2) and Equations (3a) and (4a) can now be interpreted. The Poisson effect induces lateral or secondary deformation into the phase materials in both FE Model I and II. However, in FE Model I, the phase materials can deform freely and no secondary stresses are introduced. While in FE Model II the secondary deformation is constrained by the bonded phase interface and the homogeneous boundary conditions, secondary stresses are thus induced. The development of secondary stresses and strains in FE Model II demands more strain energy to achieve the same primary deformation than in FE Model I, and therefore, the effective Young’s moduli predicted by FE Model II are generally larger than those by FE Model I. The Poisson effect is able to influence the elastic properties of the composites, the magnitude of the influence is dependent on the contrast of phase properties, especially the contrast of phase Poisson’s ratios.

In general, the Voigt formula is more accurate than the Reuss formula for the prediction of effective Young’s modulus under the respective conditions; only if the composite has a large contrast in phase Poisson’s ratios and also has similar phase Young’s moduli, the Voigt formula has low accuracy, cf. Figure 6b. It appears that the influence from a large contrast of phase Poisson’s ratios can be ‘cancelled’ by a large contrast of phase Young’s moduli, comparing Figure 6b,d. On the other hand, the Reuss formulas is accurate only if the composite has a small contrast in both of its phase Young’s moduli and phase Poisson’s ratios, cf. Figure 8a. This is probably because the iso-stress condition is more stringent than the iso-strain condition. However, neither the Voigt nor the Reuss formula is accurate for the prediction of effective Poisson’s ratio. The newly derived counterparts of the Voigt and Reuss formulas, i.e., Equations (3a)–(3c) and (4a), (4b), are able to accurately predict effective Young’s modulus and effective Poisson’s ratio under the respective conditions, due to the consideration of the Poisson effect.

It has been found that for unidirectional composites [21,22,23], the longitudinal Young’s modulus can be predicted by the Voigt formula with satisfactory accuracy, but the Reuss formula is usually inaccurate for the prediction of the transverse Young’s modulus. There are two possible reasons. One is the neglection of the Poisson effect in the formulas, and the Poisson effect has stronger influence over the Reuss formula than on the Voigt formula; the other reason is that for unidirectional composites, the iso-strain condition is satisfied by the ‘longitudinal model’, but the iso-stress condition is not satisfied by the ‘transverse model’. It can be verified by finite element modeling, the average strains in the fibers and in the matrix are equal to each other, if a uniform stretching is applied in the longitudinal direction; However, the average stresses in the fibers and in the matrix are not equal to each other when a uniform loading is applied in the transverse direction. The difference is caused by the fact that the fibers are continuous in the longitudinal direction, but not continuous in the transverse direction.

The Voigt and Reuss formulas are increasingly applied in the study of emerging nanocomposites and functionally graded materials [9,10,11,12,13,14,15,16,17,18,19,20], mainly used to determine the effective properties, either for a whole RVE or at a specific location. Since the effective properties is the base for us to understand the material behavior at the macroscopic scale, they must be accurate and reliable. The study results suggest the conventional Voigt and Reuss formulas may be inaccurate because the Poisson effect is not considered. The newly developed formulas, Equations (3a)–(3c) and (4a), (4b), can be applied in replacement of the conventional formulas for the study of novel composite materials. The study results presented in Figure 6, Figure 7, Figure 8 and Figure 9 may also serve as a guide to judge the accuracy in case that the Voigt and Reuss formulas are still preferred due to their simplicity.

## 5. Conclusions

The analytical derivation and the numerical results show that the conventional Voigt and Reuss formulas are special cases of the newly derived counterparts, the Voigt formula and especially the Reuss formula are accurate for effective Young’s modulus only under the special conditions that can devoid or diminish the Poisson effect; neither of the formulas is accurate for the prediction of effective Poisson’s ratio. The physical mechanism is, the Poisson effect induces secondary strains and stresses into the phase materials, which demands more strain energy to achieve the same deformation in the loading (primary) direction, and thus increase composite stiffness. The increase in composite stiffness is dependent on the contrasts of phase properties, the contrast of phase Poisson’s ratios is more influential than that of phase Young’s moduli. The newly derived formulas, i.e., Equations (3a)–(3c) and (4a), (4b), can be used in the replacement of the conventional Voigt and Reuss formulas for the prediction of composite elastic properties under the iso-strain and the iso-stress conditions. The above findings may have significant impact on the study of emerging nanocomposites and functionally graded materials, where the Voigt and Reuss formulas have wide applications.

## Figures and Tables

**Table 1:** Symbols of the involved parameters and variables.

Symbol	Meaning
$E_{i}$ (i=1,2)	Young’s modulus of Phase i
$\nu_{i}$ (i=1,2)	Poisson’s ratio of Phase i
$f_{i}$ (i=1,2)	Volume fraction of Phase i
$\varepsilon_{x}^{i}$ $,\;\varepsilon_{y}^{i}$ $,\;\varepsilon_{z}^{i}$ (i=1,2)	Strain components in Phase i
$\sigma_{x}^{i}$ $,\;\sigma_{y}^{i}$ $,\;\sigma_{z}^{i}$ (i=1,2)	Stress components in Phase i
${\bar{\varepsilon }}_{x}$ $,\;{\bar{\varepsilon }}_{y}$ $,\;{\bar{\varepsilon }}_{z}$	Average strain components in the RVE
${\bar{\sigma }}_{x}$ $,\;{\bar{\sigma }}_{y}$ $,\;{\bar{\sigma }}_{z}$	Average stress components in the RVE
$V_{i}$(i=1,2)	The volume of Phase i

**Table 2:** RVE boundary conditions for the characterization of effective properties.

RVE Surface	Iso-Strain [Figure 1b]		Iso-Stress [Figure 1c]	
	${\bar{E}}_{x}$ $,\;{\bar{\nu }}_{xy}$ $,\;{\bar{\nu }}_{xz}$		${\bar{E}}_{z}$ $,\;{\bar{\nu }}_{zx}={\bar{\nu }}_{zy}$	
	HBC	IBC	HBC	IBC
x=0	$u_{x}=0$	$u_{x}=0$	$u_{x}=0$	$u_{x}=0$
y=0	$u_{y}=0$	$u_{y}=0$	$u_{y}=0$	$u_{y}=0$
z=0	$u_{z}=0$	$u_{z}=0$	$u_{z}=0$	$u_{z}=0$
x=L	$u_{x}=1$	$u_{x}=1$	$\mathrm{Homogeneous}\;u_{x}$	Free
y=L	$\mathrm{Homogeneous}\;u_{y}$ *	Free	$\mathrm{Homogeneous}\;u_{y}$	Free
z=L	$\mathrm{Homogeneous}\;u_{z}$	Free	$u_{z}=1$	$u_{z}=1$

* All nodes on the surface y=L are forced to have the same displacement $u_{y}$.

**Table 3:** Two-phase composites with different contrasts of phase properties.

Composite #	Softer Phase		Stiffer Phase		Phase Contrast of	
	Young’s Modulus (MPa)	Poisson’s Ratio	Young’s Modulus (MPa)	Poisson’s Ratio	Young’s Modulus	Poisson’s Ratio
1	80.0	0.20	120.0	0.15	Small	Small
2	80.0	0.45	120.0	0.15	Small	Large
3	80.0	0.20	12,000.0	0.15	Large	Small
4	80.0	0.45	12,000.0	0.15	Large	Large

**Figure A1:**
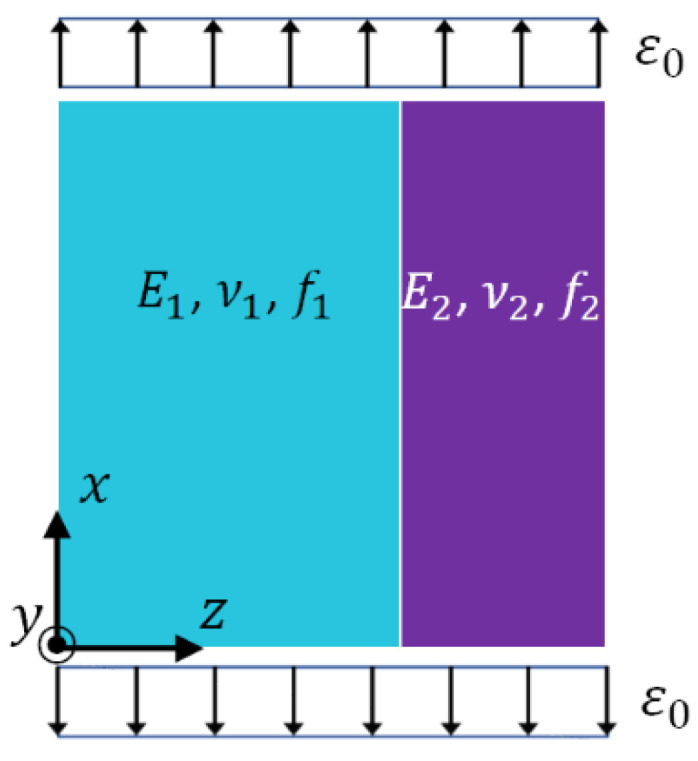
Iso-strain condition.

**Figure A2:**
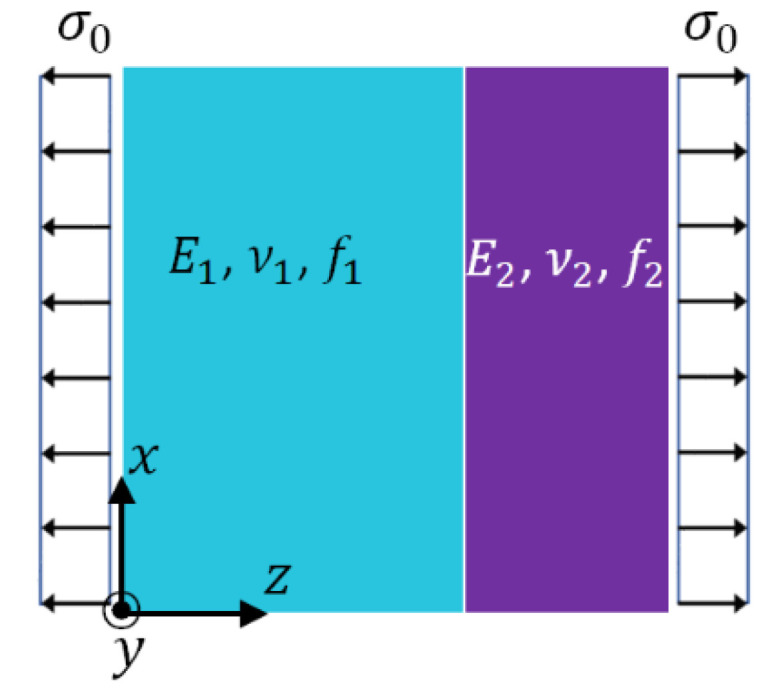
Iso-stress conditions.

## Data Availability

Not applicable.

## Appendix A

The formulas of effective Young’s modulus and effective Poisson’s ratio under the iso-strain and the iso-stress conditions with the consideration of the Poisson effect are derived from the elasticity equations, by following a similar development in [24]. The parameters and variables involved in the derivation are listed in Table 1.

Figure A1 illustrates the iso-strain condition imposed on the composite RVE. Uniform tension or compression is applied at the top and the bottom surface, so that the phase materials experience the same strain ($\varepsilon_{0}$) in the x direction.

Relations between the normal strains and the normal stresses in phase 1 and phase 2 are established from the constitutive equations of isotropic material, as shown in Equations (A1)–(A3) and Equations (A4)–(A6).

(A1) $$\varepsilon_{x}^{1}=\frac{1}{E_{1}}\left(\sigma_{x}^{1}-\nu_{1}\sigma_{y}^{1}-\nu_{1}\sigma_{z}^{1}\right)$$

(A2) $$\varepsilon_{y}^{1}=\frac{1}{E_{1}}\left(\sigma_{y}^{1}-\nu_{1}\sigma_{x}^{1}-\nu_{1}\sigma_{z}^{1}\right)$$

(A3) $$\varepsilon_{z}^{1}=\frac{1}{E_{1}}\left(\sigma_{z}^{1}-\nu_{1}\sigma_{x}^{1}-\nu_{1}\sigma_{y}^{1}\right)\;$$

(A4) $$\varepsilon_{x}^{2}=\frac{1}{E_{2}}\left(\sigma_{x}^{2}-\nu_{2}\sigma_{y}^{2}-\nu_{2}\sigma_{z}^{2}\right)$$

(A5) $$\varepsilon_{y}^{2}=\frac{1}{E_{2}}\left(\sigma_{y}^{2}-\nu_{2}\sigma_{x}^{2}-\nu_{2}\sigma_{z}^{2}\right)$$

(A6) $$\varepsilon_{z}^{2}=\frac{1}{E_{2}}\left(\sigma_{z}^{2}-\nu_{2}\sigma_{x}^{2}-\nu_{2}\sigma_{y}^{2}\right)$$

Imposition of equilibrium condition in, respectively, x, y and z direction produces the following relations between average stresses in the RVE and phase stresses.

(A7) $${\bar{\sigma }}_{x}=f_{1}\sigma_{x}^{1}+f_{2}\sigma_{x}^{2}$$

(A8) $${\bar{\sigma }}_{y}=f_{1}\sigma_{y}^{1}+f_{2}\sigma_{y}^{2}=0$$

(A9) $${\bar{\sigma }}_{z}=\sigma_{z}^{1}=\sigma_{z}^{2}=0$$

The following relations between average and phase strains are obtained by imposing kinematic constraints.

(A10) $${\bar{\varepsilon }}_{x}=\varepsilon_{x}^{1}=\varepsilon_{x}^{2}=\varepsilon_{0}$$

(A11) $${\bar{\varepsilon }}_{y}=\varepsilon_{y}^{1}=\varepsilon_{y}^{2}$$

(A12) $${\bar{\varepsilon }}_{z}=f_{1}\varepsilon_{z}^{1}+f_{2}\varepsilon_{z}^{2}$$

Equations (A1)–(A6), (A8)–(A11) are solved, favorably using a symbolic computational software such as MAPLE or MATHEMATICA, for the 12 variables $\varepsilon_{x}^{1}$, $\varepsilon_{y}^{1}$, $\varepsilon_{z}^{1}$, $\sigma_{x}^{1}$, $\sigma_{y}^{1}$, $\sigma_{z}^{1}$, $\varepsilon_{x}^{2}$, $\varepsilon_{y}^{2}$, $\varepsilon_{z}^{2}$, $\sigma_{x}^{2}$, $\sigma_{y}^{2}$ and $\sigma_{z}^{2}$. It should be noticed that each of Equations (A9) and (A10) contains two equations. The solved variables are then substituted into Equations (A7) and (A12) for the determination of ${\bar{\sigma }}_{x}$ and ${\bar{\varepsilon }}_{z}$. Iso-strain formula of the effective Young’s modulus of the RVE is derived as

(A13) $${\bar{E}}_{\mathrm{iso}-\mathrm{strain}}={\bar{E}}_{x}=\frac{{\bar{\sigma }}_{x}}{{\bar{\varepsilon }}_{x}}=\;\frac{\left[f_{1}\left(1-\nu_{2}\right)E_{1}+f_{2}\left(1-\nu_{1}\right)E_{2}\right]\cdot \left[f_{1}\left(1+\nu_{2}\right)E_{1}+f_{2}\left(1+\nu_{1}\right)E_{2}\right]}{f_{1}\left(1-\nu_{2}^{2}\right)E_{1}+f_{2}\left(1-\nu_{1}^{2}\right)E_{2}}$$

The average transverse strains in y and z direction, i.e., ${\bar{\varepsilon }}_{y}$ and ${\bar{\varepsilon }}_{z}$, are not the same. Therefore, two Poisson’s ratios, ${\bar{\nu }}_{xy}$ and ${\bar{\nu }}_{xz}$, are required to describe the transverse deformation of the RVE.

(A14) $${\bar{\nu }}_{\mathrm{iso}-\mathrm{strain}}^{P}={\bar{\nu }}_{xy}=-\frac{{\bar{\varepsilon }}_{y}}{{\bar{\varepsilon }}_{x}}=\;\frac{\left[f_{1}\nu_{1}\left(1-\nu_{2}\right)+f_{2}\nu_{2}\left(1-\nu_{1}\right)\right]\cdot \left[f_{1}\left(1+\nu_{2}\right)E_{1}+f_{2}\left(1+\nu_{1}\right)E_{2}\right]}{f_{1}\left(1-\nu_{2}^{2}\right)E_{1}+f_{2}\left(1-\nu_{1}^{2}\right)E_{2}}$$

(A15) $${\bar{\nu }}_{\mathrm{iso}-\mathrm{strain}}^{T}={\bar{\nu }}_{xz}=-\frac{{\bar{\varepsilon }}_{z}}{{\bar{\varepsilon }}_{x}}=\;\frac{f_{1}\nu_{1}\left(1-\nu_{2}^{2}\right)E_{1}+f_{2}\nu_{2}\left(1-\nu_{1}^{2}\right)E_{2}}{f_{1}\left(1-\nu_{2}^{2}\right)E_{1}+f_{2}\left(1-\nu_{1}^{2}\right)E_{2}}$$

Iso-stress formulas can be derived in a similar way. Figure A2 shows that the iso-stress condition is applied to the composite RVE. The strain-stress relations in the phase materials are the same as those established in Equations (A1)–(A6). Relations between RVE average and phase stresses (or strains) are also obtained by imposing iso-stress equilibrium and kinematic conditions on the RVE. The resulting equations are provided in Equations (A16)–(A18) and Equations (A19)–(A21), respectively.

(A16)
$${\bar{\sigma }}_{x}=f_{1}\sigma_{x}^{1}+f_{2}\sigma_{x}^{2}=0$$

(A17)
$${\bar{\sigma }}_{y}=f_{1}\sigma_{y}^{1}+f_{2}\sigma_{y}^{2}=0$$

(A18)
$${\bar{\sigma }}_{z}=\sigma_{z}^{1}=\sigma_{z}^{2}=\sigma_{0}$$

(A19)
$${\bar{\varepsilon }}_{x}=\varepsilon_{x}^{1}=\varepsilon_{x}^{2}$$

(A20)
$${\bar{\varepsilon }}_{y}=\varepsilon_{y}^{1}=\varepsilon_{y}^{2}$$

(A21)
$${\bar{\varepsilon }}_{z}=f_{1}\varepsilon_{z}^{1}+f_{2}\varepsilon_{z}^{2}$$

Solve Equations (A1)–(A6), (A16)–(A20) for the 12 variables $\varepsilon_{x}^{1}$, $\varepsilon_{y}^{1}$, $\varepsilon_{z}^{1}$, $\sigma_{x}^{1}$, $\sigma_{y}^{1}$, $\sigma_{z}^{1}$, $\varepsilon_{x}^{2}$, $\varepsilon_{y}^{2}$, $\varepsilon_{z}^{2}$, $\sigma_{x}^{2}$, $\sigma_{y}^{2}$ and $\sigma_{z}^{2}$. The solutions are then used to determine the effective Young’s modulus and the effective Poisson’s ration of the RVE.

(A22) $${\bar{E}}_{\mathrm{iso}-\mathrm{stress}}={\bar{E}}_{z}=\frac{{\bar{\sigma }}_{z}}{{\bar{\varepsilon }}_{z}}=\;\frac{E_{1}E_{2}\left[f_{1}\left(1-\nu_{2}\right)E_{1}+f_{2}\left(1-\nu_{1}\right)E_{2}\right]}{E_{1}E_{2}\left[f_{1}^{2}\left(1-\nu_{2}\right)+f_{2}^{2}\left(1-\nu_{1}\right)\right]+f_{1}f_{2}\left[\left(1+\nu_{2}\right)\left(1-2\nu_{2}\right)E_{1}^{2}+4\nu_{1}\nu_{2}E_{1}E_{2}+\left(1+\nu_{1}\right)\left(1-2\nu_{1}\right)E_{2}^{2}\right]}\;$$

Since the x-y plane is the so-called isotropic plane, the average strain in the x and y direction, i.e., ${\bar{\varepsilon }}_{x}$ and ${\bar{\varepsilon }}_{y}$, are theoretically the same. Therefore, Poisson’s ratios in the two directions are equal, i.e.,

(A23) $${\bar{\nu }}_{\mathrm{iso}-\mathrm{stress}}={\bar{\nu }}_{zx}={\bar{\nu }}_{zy}=-\frac{{\bar{\varepsilon }}_{y}}{{\bar{\varepsilon }}_{z}}=\;\frac{E_{1}E_{2}\left[f_{1}\left(1-\nu_{2}\right)\nu_{1}+f_{2}\left(1-\nu_{1}\right)\nu_{2}\right]}{E_{1}E_{2}\left[f_{1}^{2}\left(1-\nu_{2}\right)+f_{2}^{2}\left(1-\nu_{1}\right)\right]+f_{1}f_{2}\left[\left(1+\nu_{2}\right)\left(1-2\nu_{2}\right)E_{1}^{2}+4\nu_{1}\nu_{2}E_{1}E_{2}+\left(1+\nu_{1}\right)\left(1-2\nu_{1}\right)E_{2}^{2}\right]}\;$$

It should be pointed out that, in finite element modeling of the RVE, Equation (A11) in the iso-strain condition and Equations (A19) and (A20) in the iso-stress condition, are implemented as homogeneous boundary conditions.
